# Transcriptomic insights into pseudorabies virus suppressed cell death pathways in neuroblastoma cells

**DOI:** 10.3389/fmicb.2024.1430396

**Published:** 2024-09-19

**Authors:** Shinuo Cao, Li Zhang, Mo Zhou, Shanyuan Zhu

**Affiliations:** Swine Infectious Diseases Division, Jiangsu Key Laboratory for High-Tech Research and Development of Veterinary Biopharmaceuticals, Engineering Technology Research Center for Modern Animal Science and Novel Veterinary Pharmaceutic Development, Jiangsu Agri-animal Husbandry Vocational College, Taizhou, China

**Keywords:** pseudorabies virus, Neuro-2a cells, transcriptomics, inflammatory, cell death

## Abstract

Pseudorabies virus (PRV) exhibits a complex interplay of host-pathogen interactions, primarily by modulating host cell death pathways to optimize its replication and spread in Neuro-2a cells. Using high-throughput RNA sequencing, we identified 2,382 upregulated differentially expressed genes (DEGs) and 3,998 downregulated DEGs, indicating a intricate interaction between viral pathogenesis and host cellular responses. This research offers valuable insights into the molecular processes involved in PRV infection, highlighting the substantial inhibition of crucial cell death pathways in Neuro-2a cells, including necroptosis, pyroptosis, autophagy, ferroptosis, and cuproptosis. Cells infected with PRV exhibit decreased expression of genes critical in these pathways, potentially as a mechanism to avoid host immune reactions and ensure cell survival to support ongoing viral replication. This extensive inhibition of apoptosis and metabolic alterations highlights the sophisticated tactics utilized by PRV, enhancing our comprehension of herpesvirus biology and the feasibility of creating specific antiviral treatments. This research contributes to our understanding of how viruses manipulate host cell death and presents potential opportunities for therapeutic interventions to disrupt the virus’s lifecycle.

## Introduction

Pseudorabies virus (PRV), belonging to the family *Herpesviridae* and subfamily *Alphaherpesvirinae*, has been recognized as the etiological agent of Aujeszky’s disease, which presents with a broad spectrum of symptoms ranging from respiratory illness to neurological disorders in pigs and other animals (Pomeranz et al., 2005; Davison et al., 2009). Herpesvirus genomes can be divided into six classes according to the arrangement of repeat sequences and unique regions. As a linear, double-stranded, sense viral DNA genome of approximately 145 kb, PRV belongs to the D class, with a 74% G + C content (Romero et al., 1997; Pomeranz et al., 2005). It was reported in 2017 that PRV had infected the first human in mainland China (Ai et al., 2018; Yang et al., 2019; Liu et al., 2021). When PRV infects an adult pig, its primary replication takes place in the respiratory epithelium before it enters peripheral nerve endings, which include those in the sensory trigeminal ganglia and olfactory bulb (Babic et al., 1994). PRV particles can retrogradely transport to sensory and autonomic peripheral ganglia, establishing a reactivable latent infection. PRV can maintain a lifelong latent infection in peripheral nervous system neurons without clinical symptoms post-recovery. Stress-induced reactivation causes viral replication in peripheral nervous system ganglia and anterograde spread to mucosal surfaces, resulting in mild respiratory signs (Maes et al., 1983). Peripheral nervous system rarely reaches the pregnant uterus and the central nervous system to cause encephalitis but can spread to uninfected pigs, accumulating in populations (Tirabassi et al., 1998).

The Neuro-2a cell line, derived from neuroblastoma in mice, has been widely utilized in research to investigate processes such as neuronal differentiation, axonal growth, and signaling (Tremblay et al., 2010). The Neuro-2a cell line’s ability to undergo differentiation into neuron-like cells in suitable environments renders them a valuable model for investigating the mechanisms involved in viral neurotropism and the neurological impairments. Observed *in vitro* (Dickey et al., 2004). This correlation has played a crucial role in offering insights into the neurodegenerative mechanisms initiated by viral infections (Andsberg et al., 2002). Transcriptomics has become an essential tool for molecular biologists, especially in infectious diseases, enabling a comprehensive analysis of changes in gene expression induced by pathogens. This thorough profiling assists in elucidating the temporal dynamics of interactions between hosts and pathogens, identifying differentially expressed genes, and comprehending the active regulatory networks throughout the course of infection.

The neuropathological effects encompass a cascade of molecular reactions leading to cell death, inflammation, disruption of intracellular signaling pathways, and metabolic alterations (Jellinger, 2010; Salucci et al., 2021; Li et al., 2024). The apoptotic machinery is often co-opted by virus to facilitate its replication and spread, while simultaneously eluding the host’s immune surveillance (Marianneau et al., 1998; Rex et al., 2022). Neuro-2a cell line may provide insight into the molecular complexities of apoptosis induced by PRV, encompassing the analysis of intrinsic and extrinsic pathways. Similarly, the capacity of PRV infection to elicit inflammatory responses via the release of cytokines and chemokines plays a crucial role in the pathogenesis of the infection (Sun et al., 2023; Zhou N. et al., 2023; Zhou Q. et al., 2023). The virus alters intracellular signaling cascades, including the MAPK, NF-κB, and PI3K/Akt pathways, that regulate cell survival, proliferation, and stress responses (Laval et al., 2019). The metabolic reprogramming observed during PRV infection indicates the virus’s utilization of the host’s biosynthetic and energy-producing mechanisms to facilitate its life cycle. The utilization of transcriptomics in these investigations offers a comprehensive view of the metabolic genes and pathways altered by PRV, enhancing understanding of the virus’s influence on host cell metabolism.

In this study, transcriptomic analyses of PRV infection in Neuro-2a cells have the potential to elucidate the intricate network of host reactions and reveal the molecular tactics utilized by the virus for proliferation within the nervous system. Utilizing a transcriptomic methodology is crucial in elucidating the complexities of PRV-induced neuropathogenesis, thereby enhancing the efficacy of therapeutic strategies against this formidable pathogen. The insights gleaned from these studies are confined to PRV and extend to a broader understanding of herpesvirus biology and the general principles of viral neurotropism.

## Materials and methods

### RNA extraction, library construction, and sequencing

Total RNA was isolated using the Trizol reagent kit (Invitrogen, Carlsbad, CA, United States) as per the manufacturer’s instructions. The quality of the RNA was evaluated using an Agilent 2100 Bioanalyzer (Agilent Technologies, Palo Alto, CA, United States) and verified via RNase-free agarose gel electrophoresis. Post extraction, eukaryotic mRNA was enriched utilizing Oligo(dT) beads, whereas prokaryotic mRNA enrichment was achieved by depleting rRNA using the Ribo-Zero™ Magnetic Kit (Epicentre, Madison, WI, United States). Subsequently, the enriched mRNA was fragmented into smaller pieces using fragmentation buffer and reverse transcribed into cDNA with random primers. The synthesis of the second-strand cDNA was carried out using DNA polymerase I, RNase H, dNTPs, and buffer. Following this, the cDNA fragments underwent purification with the QiaQuick PCR extraction kit (Qiagen, Venlo, The Netherlands), end-repair, poly(A) tailing, and ligation to Illumina sequencing adapters. The ligation products were size-selected through agarose gel electrophoresis, PCR amplified, and sequenced on an Illumina HiSeq2500 platform by Gene Denovo Biotechnology Co. (Guangzhou, China). Reads obtained from the sequencing machines include raw reads with adapters or low-quality bases that can negatively impact subsequent assembly and analysis. Therefore, to ensure high-quality clean reads, the raw reads were further processed and filtered using fastp (version 0.18.0).

### Data preprocessing and differential gene expression analysis

Differential expression analysis was conducted utilizing the DESeq2 software between two different groups (and by edgeR between two samples), with genes deemed significantly differentially expressed at an adjusted *p*-value of less than 0.05 and an absolute log2 fold change of at least 1 (Robinson et al., 2010; Love et al., 2014).

### Principal component analysis

Principal component analysis (PCA) was performed with R package gmodels^1^ in this experience. PCA is a statistical procedure that converts hundreds of thousands of correlated variables (gene expression) into a set of values of linearly uncorrelated variables called principal components. PCA is largely used to reveal the structure/relationship of the samples/datas.

### Gene ontology and pathway enrichment analysis

Gene Ontology (GO) is an internationally standardized system for gene functional classification, providing a dynamic and updated controlled vocabulary and well-defined concepts to describe gene properties and their products across any organism (Ashburner et al., 2000). GO encompasses three ontologies: molecular function, cellular component, and biological process. Each basic unit in GO is a GO-term, categorized into one of the ontologies. GO enrichment analysis identifies GO terms significantly enriched in differentially expressed genes (DEGs) compared to the genomic background, thereby highlighting the biological functions associated with DEGs. Initially, all DEGs were mapped to GO terms in the Gene Ontology database,^2^ and gene numbers were calculated for each term. Significantly enriched GO terms were identified using a hypergeometric test by comparing the DEG count to the genomic background. The resulting *p*-values were adjusted using FDR correction, with FDR ≤ 0.05 as the threshold for significance. GO terms meeting this criterion were considered significantly enriched. This analysis elucidates the primary biological functions of the DEGs.

### Expression analysis of cell death pathways

Volcano plots were employed to identify and visualize DEGs. Pyroptosis-related genes (PRGs) were sourced from the Molecular Signatures Database (MsigDB).^3^ The necroptosis-related genes (NRGs) are referenced in the publication by Zhang et al. (2023). The AUTOPHAGY DATABASE and THANATOS^4^ provided the dataset of autophagy-related genes (ARGs). From FerrDB,^5^ a collection of 144 confirmed genes associated with ferroptosis (FRGs) was gathered (Zhou N. et al., 2023; Zhou Q. et al., 2023). The cuproptosis-related genes (CRGs) were referenced according to the publication by Tsvetkov et al. (2022). The disulfidptosis-related genes (DRGs) were referenced according to the publication by Liu et al. (2023). The analysis focused on PRGs, NRGs, ARGs, FRGs, CRGs, and DRGs that showed differential expression by intersecting DEGs with ARGs, and their expression patterns were further examined using heatmaps.

### Analysis of cytokine expression and immune pathway activation

The immunological response of PAMs to PRV infection was analyzed through gene expression profiling. Heatmaps were generated to visualize the expression levels of genes involved in the KEGG_CYTOKINE_CYTOKINE_RECEPTOR_INTERACTION (M4060) for cytokine activity and the REACTOME_CYTOSOLIC_SENSORS_OF_PATHOGEN_DNA (M5100) for detecting cytosolic DNA. The cytokine pathway included 183 genes, whereas the DNA sensing pathway contained 49 genes.

### Identification of hub genes

The process of identifying central genes within a network involves recognizing those with significant connections within specified gene clusters. This was achieved by assessing the strength of gene-to-module relationships using Pearson’s correlation coefficient, setting a threshold of |cor.geneModuleMembership| greater than 0.8. Key gene groups were then analyzed using the STRING database for constructing protein–protein interaction (PPI) networks, selecting a minimum confidence score of 900. Genes with a connectivity degree of eight or more were classified as central genes in the PPI network.

All data generated and analyzed from this study are included in this published article. The raw RNA-Seq data has been submitted to NCBI Short Read Archive (SRA) under Bioproject PRJNA1137426 (https://www.ncbi.nlm.nih.gov/bioproject/PRJNA1137426/).

### Statistical analysis

Statistical analyses were performed using GraphPad Prism 8 (San Diego, CA, United States). Data are expressed as means ± standard deviations (SDs) from at least three separate trials. A significance threshold of *p < 0.05 was used to determine statistical significance. More stringent levels of significance were indicated by **p < 0.01 and ***p < 0.001, signifying very significant and highly significant outcomes, respectively.

## Results

### Transcriptional landscapes reveal PRV-driven distinct host gene transcription at 48 h.p.i.

**Figure 1:**
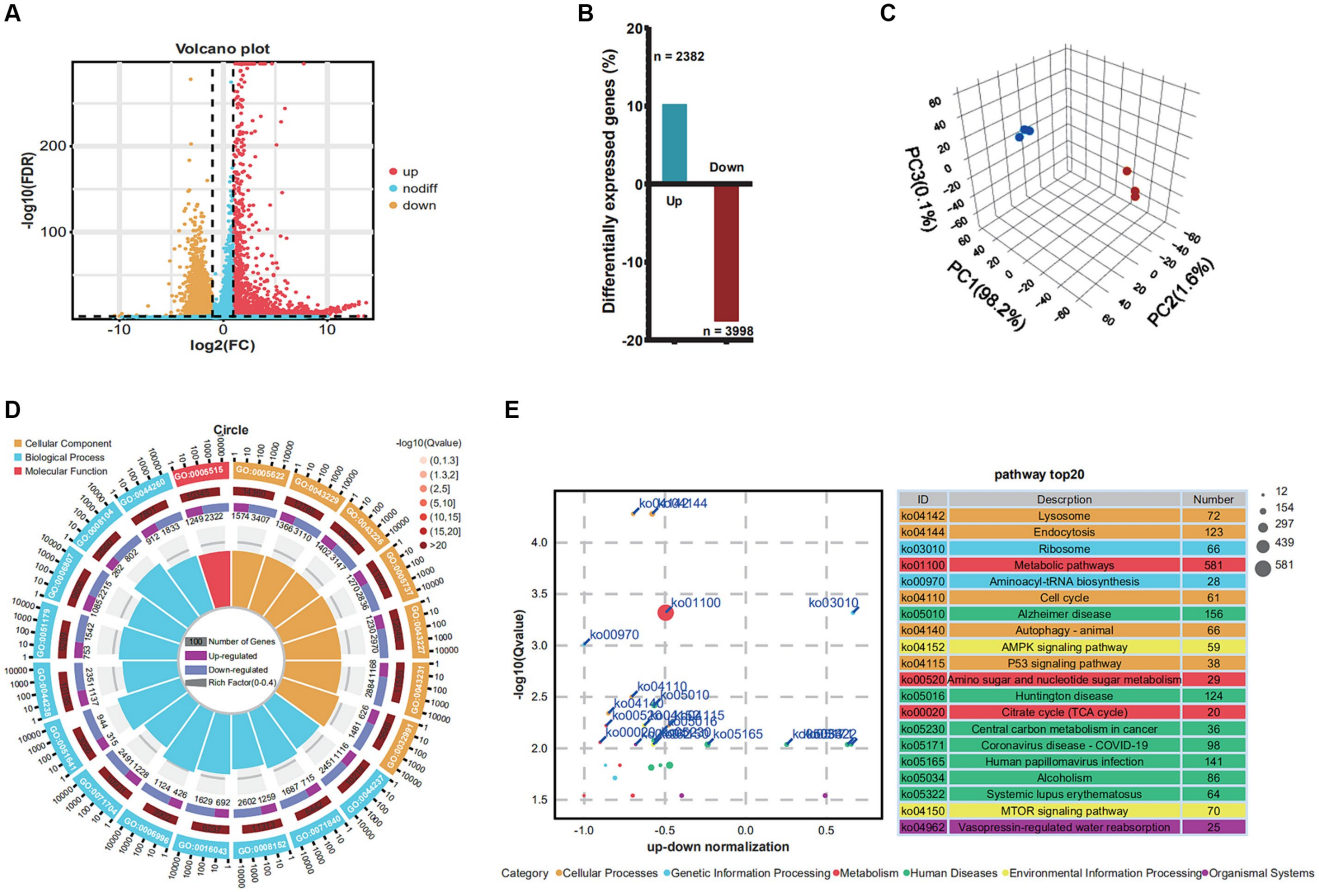
Changes in differential gene expression in Neuro-2a at 48 h.p.i. after PRV infection. **(A)** Volcano plots showing fold changes and adjusted *p*-values for genes differentially expressed between unstimulated (mock) and PRV-stimulated Neuro-2a. **(B)** The upregulated/downregulated and the total number of DEGs (≥2 FC, *p* < 0.05) between unstimulated (mock) and PRV-stimulated PAMs in the transcriptomic data at 6, 12, and 24 h, respectively. **(C)** The principal component analysis (PCA). **(D)** GO analysis of the genes with expression changes for the integrated data of the three time points. **(E)** KEGG analysis of the genes with expression changes at 48 h.p.i. and the integrated data.

To determine the impact of PRV infection on gene expression and biological processes, we analyzed differentially expressed genes (DEGs) and performed GO and KEGG enrichment analyses. Genes demonstrating a fold change of at least two-fold (FC) at 48 h post-infection (h.p.i.) were identified as differentially expressed genes (DEGs) with a significance level of *p* < 0.05, as shown in Figure 1A. The findings revealed a total of 6,380 DEGs (*p*-value < 0.05 and |log2FC| > 1) at 48 h.p.i., with 2,382 genes being upregulated and 3,998 genes being downregulated. Principal component analysis (PCA) was utilized to assess dissimilarities among the samples, as illustrated in Figure 1B. The extent of DEGs during PRV infection was determined by comparing the transcriptome profiles of the PRV-infected and mock-infected groups at various time points (Figure 1C).

In order to discern how PRV infection impacts biological processes, GO enrichment mapping was performed on DEGs. The DEGs that emerged subsequent to PRV infection were predominantly implicated in biological processes associated with the cellular metabolic process, cellular component organization or biogenesis, metabolic process, and cellular component organization, organelle organization. The cellular component category exhibited enrichment of intracellular anatomical structure, intracellular organelle, organelle, cytoplasm, and membrane-bounded organelle. The molecular function category exhibited enrichment of protein binding, binding, ion binding, catalytic activity, and heterocyclic compound binding, as depicted in Figure 1D. To explore the functional pathways of differentially expressed genes (DEGs) in Neuro-2a infected with PRV, KEGG analysis was performed on the DEGs (Figure 1E). The results revealed that the KEGG analysis exhibited enrichment of lysosome, endocytosis, ribosome, metabolic pathways, and aminoacyl-trna biosynthesis.

### PRV infection suppresses necroptosis and pyroptosis pathways

**Figure 2:**
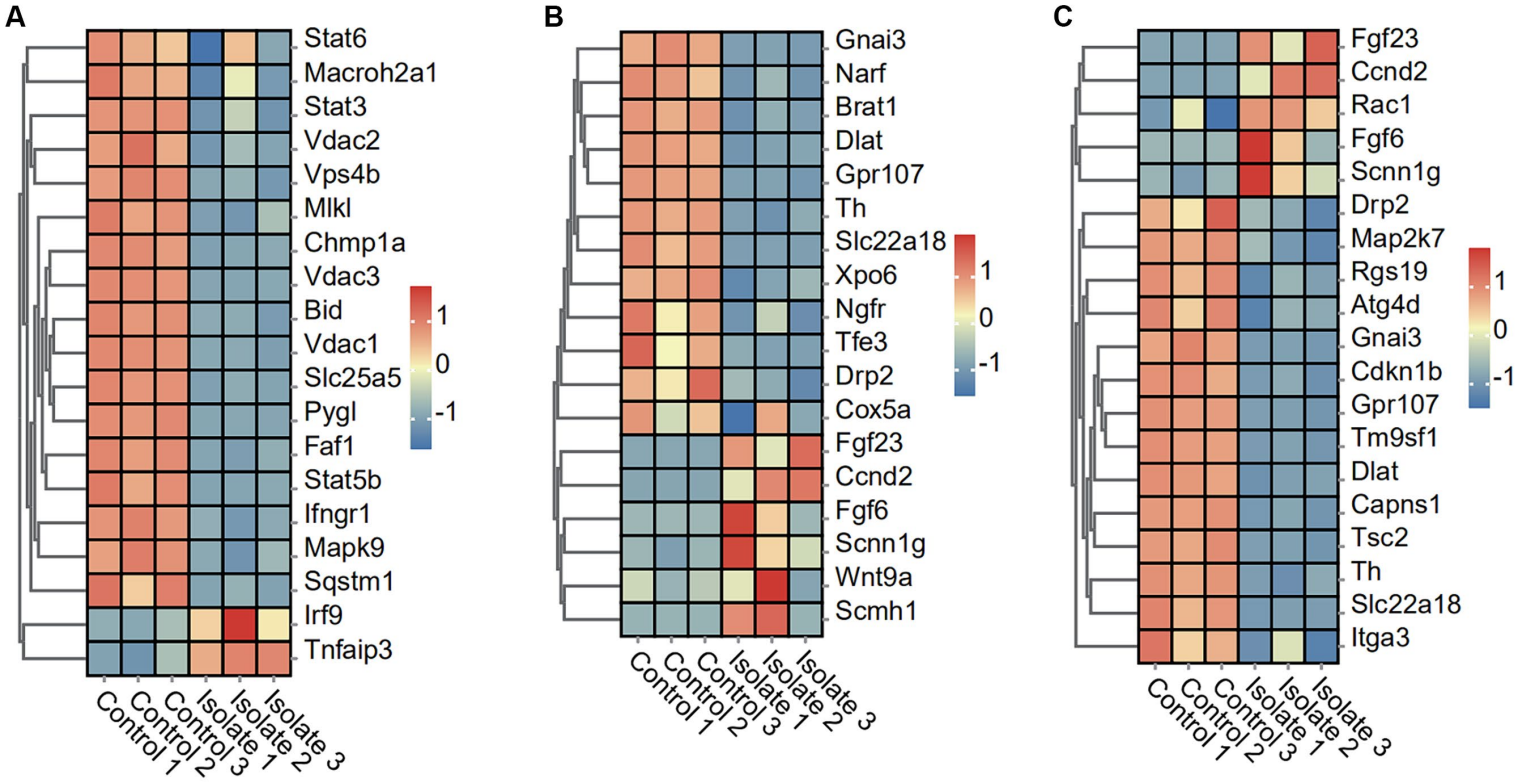
DEGs in Neuro-2a after *in vitro* PRV-specific stimulation reveal concomitant necroptosis, pyroptosis, and autophagy responses. **(A–C)** Heatmap analysis of PRV DEGs for necroptosis, pyroptosis, and autophagy post-PRV infection.

To elucidate the effects of PRV infection on necroptosis and pyroptosis pathways, we analyzed the expression profiles of related genes in Neuro-2a cells. The present study utilized the heatmap to obtain the expression profile matrix of genes related to necroptosis and pyroptosis. The findings indicate that significant expression differences exist between PRV-infected and normal samples of necroptosis and pyroptosis-related genes. Specifically, 17 necroptosis genes (Stat6, Macroh2a1, Stat3, Vdac2, Vps4b, MIk, Chmp1a, Vdac3, Bid, Vdac1, Slc25a5, Pygl, Faf1, Stat5b, lfngr1, Mapk9, and Sqstm 1) in the necroptosis-activating pathway were found to be significantly downregulated in PRV-infected neuro-2a, whereas two necroptosis genes (Irf9 and Tnfaip3) were upregulated (Figure 2A). Moreover, 12 pyroptosis genes (Gnai3, Narf, Brat1, Dlat, Gpr107, Th, Slc22a18, Xpo6, Ngfr, Tfe3, Drp2, and Cox5a) in the pyroptosis-activating pathway were found to be significantly downregulated in PRV-infected neuro-2a, whereas six pyroptosis genes (Fgf23, Ccnd2, Fgf6, Scnn1g, Wnt9a, and Scmh1) were upregulated (Figure 2B).

### PRV infection suppresses autophagy and disulfidptosis pathways

To investigate the impact of PRV infection on autophagy and disulfidptosis pathways, we analyzed the expression levels of related genes in Neuro-2a cells. The results show a significant decrease in the expression of 14 autophagy-associated genes, including Drp2, Map2k7, Rgs19, Atg4d, Gnai3, Cdkn1b, Gpr107, Tm9sf1, Dlat, Capns1, Tsc2, Th, SIc22a18, and ltga3, in neuro-2a cells infected with PRV compared to mock-infected cells. The study observed a significant upregulation in the expression of five autophagy-associated genes, including Fgf23, Ccnd2, Rac1, Fgf6, and Scnn1g, in neuro-2a cells infected with PRV compared to mock-infected cells (Figure 2C).

**Figure 3:**
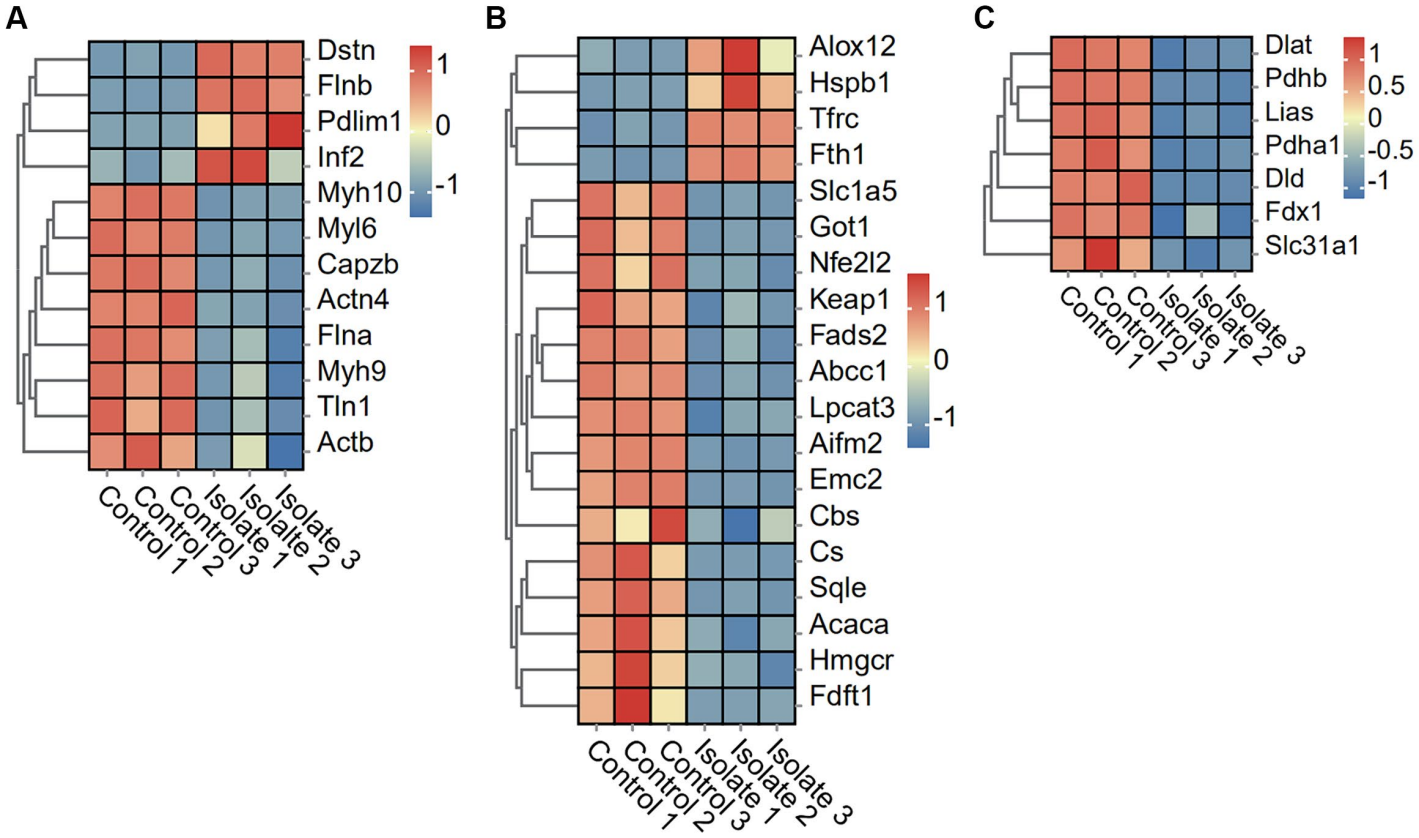
DEGs in Neuro-2a after *in vitro* PRV-specific stimulation reveal concomitant disulfidptosis, ferroptosis, and cuproptosis responses. **(A–C)** Heatmap analysis of PRV DEGs for disulfidptosis, ferroptosis, and cuproptosis post-PRV infection.

The results show a significant decrease in the expression of eight disulfide ptosis-associated genes, including Myh10, Myl6, Capzb, Actn4, FIna, Myh9, TIn1, and Actb, in neuro-2a cells infected with PRV compared to mock-infected cells. The study observed a significant upregulation in the expression of four disulfidptosis-associated genes, including Dstn, FInb, Pdlim1, and Inf2, in neuro-2a cells infected with PRV compared to mock-infected cells (Figure 3A).

### PRV infection suppresses ferroptosis and cuproptosis and pathways

To examine the effects of PRV infection on ferroptosis and cuproptosis pathways, we analyzed the differential expression of related genes in Neuro-2a cells. The PRV infection leads to a significant downregulation of 15 ferroptosis-associated genes, including Slc1a5, Got1, Nfe212, Keap1, Fads2, Abcc1, Lpcat3, Aifm2, Emc2, Cbs, Cs, Sqle, Acaca, Hmgcr, and Fdft1, when compared to mock-infected cells. Conversely, four ferroptosis-associated genes, namely Alox12, Hspb1, Tfrc, and Fth1, were found to be significantly upregulated in PRV-infected neuro-2a cells as compared to mock-infected cells (Figure 3B). In addition, the PRV infection leads to a significant downregulation of seven cuproptosis-associated genes, including Dlat, Pdhb, Lias, Pdha1, Dld, Fdx1, and Slc31a1, when compared to mock-infected cells (Figure 3C).

### Transcriptomic profiles of inflammatory-related genes responding to PRV infection

**Figure 4:**
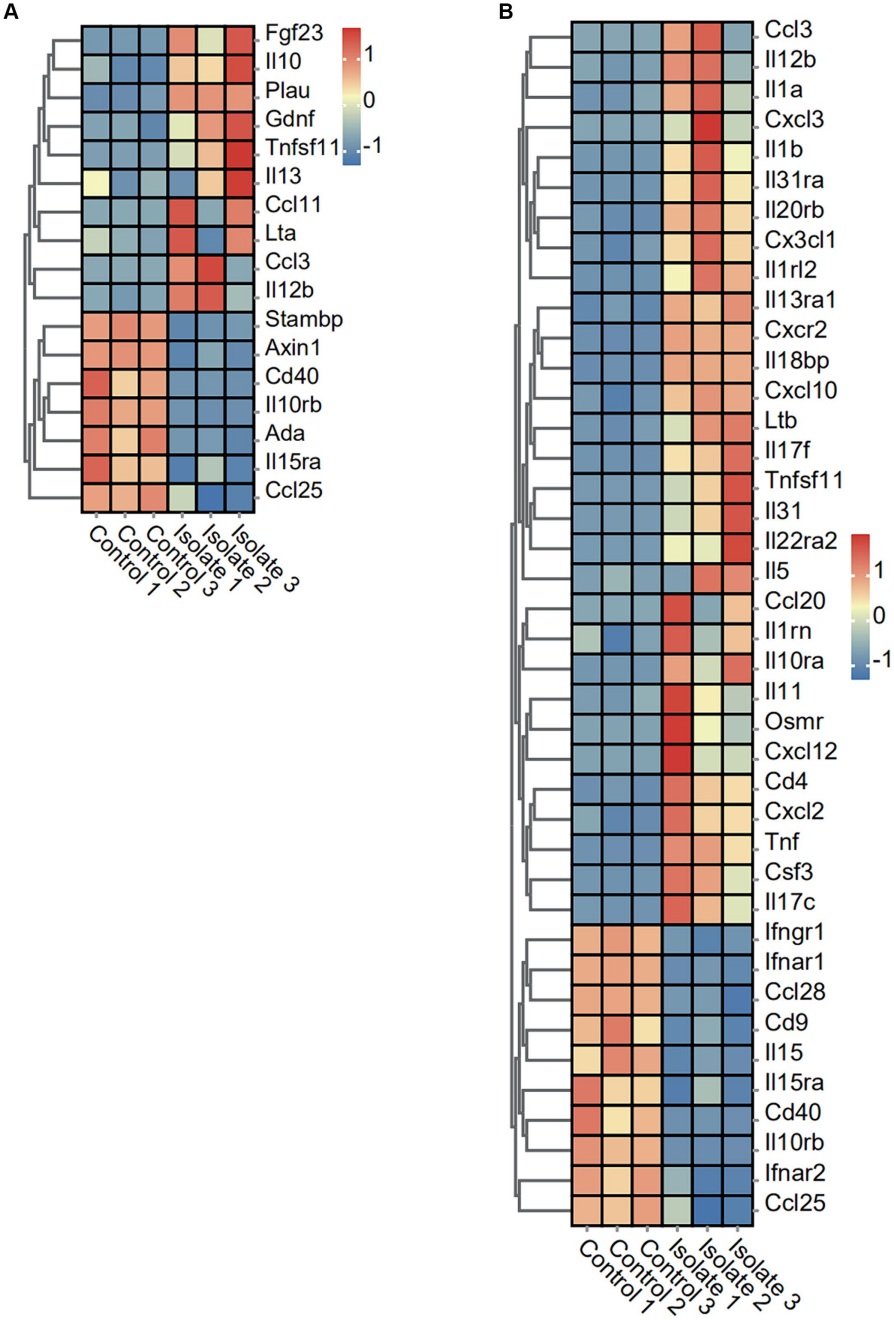
Effect of PRV infection on the transcription of anti-viral factors, cytokines, chemokines, and inflammatory associated genes in PRV infected PAMs. **(A)** Heatmap analysis of PRV DEGs for inflammation post-PRV infection. **(B)** Heatmap analysis of PRV DEGs for cytokines and chemokines post-PRV infection.

To investigate the impact of PRV infection on inflammatory response markers, we analyzed the expression levels of various cytokines and chemokines. The expression of 10 inflammatory response markers (Fgf23, IL10, Plau, Gdnf, Tnfsf11, IL13, Ccl11, Lta, Ccl3, and IL12b) was significantly induced by PRV infection, while the expression of Stambp, Axin1, Cd40, IL10rb, Ada, IL15ra, and Ccl25 was suppressed (Figure 4A). Moreover, the expression of 29 chemokine and cytokine markers (Ccl3, IL12b, IL1a, Cxcl3, IL1b, IL31ra, IL20rb, Cx3cl1, IL1rl2, IL13ra1, Cxcr2, IL18bp, Cxcl10, Ltb, Tnfsf11, IL31, IL22ra2, IL5, Ccl20, IL1rn, IL10ra, IL11, Osmr, Cxcl12, Cd4, Cxc12, Tnf, Csf3, and IL17c) was significantly induced by PRV infection, while the expression of Ifngr1, lfnar1, Ccl28, Cd9, IL15, IL15ra, Cd40, IL10rb, lfnar2, and Ccl25 was suppressed (Figure 4B).

### Screening of molecular markers associated with PRV immunopathology based on machine learning methods

**Figure 5:**
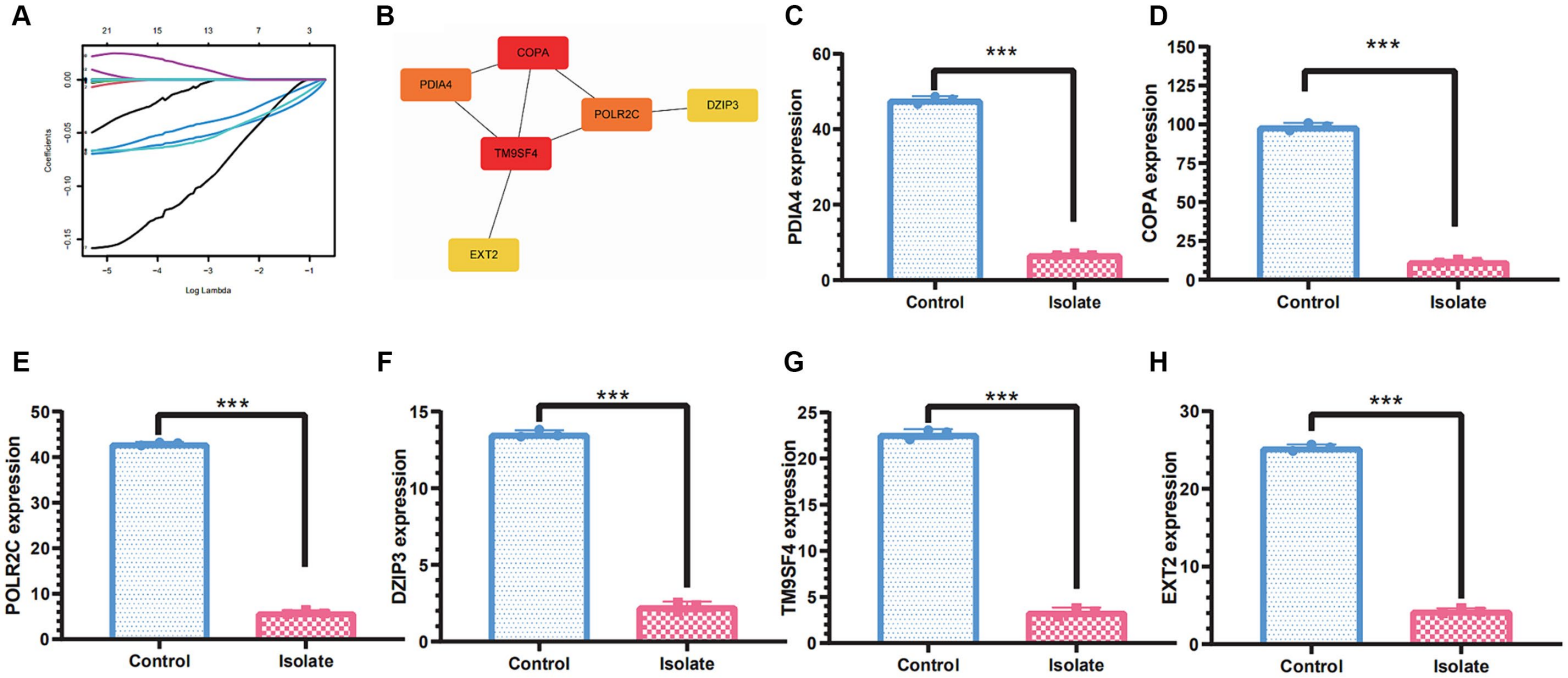
Molecular markers associated with PRV immunopathology based on machine learning methods. **(A)** Least absolute shrinkage and selection operator (LASSO) regression with 10-fold cross-validation was used to reduce the dimension of the grouping characteristics. **(B)** PPI network of the molecular markers including PDIA4, COPA, POLR2C, DZIP3, TM9SF4, and EXT2. **(C–H)** The molecular markers including PDIA4, COPA, POLR2C, DZIP3, TM9SF4, and EXT2 were significantly downregulated in PRV-infected cells than in mock-infected cells. A significance threshold of *p<0.05 was used to determine statistical significance, with more stringent levels indicated by **p<0.01 and ***p<0.001, signifying very significant and highly significant outcomes, respectively.

To identify molecular markers associated with PRV immunopathology, we employed machine learning techniques to pinpoint potential biomarkers for PRV infection and develop genetic diagnostic tools. Machine learning techniques can be used to identify biomarkers for PRV infection and diagnose the infection genetically. The data dimensions were reduced using a software defect prediction (SDP) model based on support vector machines (SVMs) and least absolute shrinkage and selection operators (LASSOs). Ultimately, the six genes, namely PDIA4, COPA, POLR2C, DZIP3, TM9SF4, and EXT2, were identified as feature genes. In PRV-infected cells, the expression levels of these five feature genes were significantly lower than in mock-infected cells based on quantifiable transcriptomic data (Figure 5).

## Discussion

Our results provide compelling evidence that PRV infection leads to a comprehensive suppression of several key cell death pathways. The suppression of genes associated with necroptosis and pyroptosis indicates a deliberate viral intervention aimed at evading host immune responses, particularly those of a pro-inflammatory nature that may result in the swift elimination of infected cells. This suppression functions to preserve the viability of host cells, thereby establishing a reservoir for ongoing viral replication. The significant downregulation of genes like Stat3 and Vdac2, key mediators in necroptosis, points to a viral strategy to inhibit cell death that would otherwise limit infection spread. The observed downregulation of autophagy-related genes emphasizes PRV’s tactic of enhancing its intracellular milieu to facilitate replication. Autophagy typically serves as a cellular defense mechanism to eliminate pathogens (Levine and Kroemer, 2008, 2019). The inhibition of autophagy by PRV may impede the degradation of viral components, facilitating viral assembly and egress. Although autophagy is commonly perceived as a mechanism for cell survival, its dual function in cell death highlights the intricate nature of host-pathogen interactions, where the interplay between cell survival and death pathways influences the course of infection (Glick et al., 2010; Das et al., 2012).

Previous studies on other herpesviruses, such as Feline herpesvirus type 1 (FeHV-1), have demonstrated that these viruses induce both apoptosis and autophagy in CRFK cells. Notably, cleavage of caspase-3 significantly increased 48 h post-infection, while caspase-9 activation commenced 24 h post-infection, suggesting that mitochondrial damage plays a pivotal role. Additionally, FeHV-1 infection markedly induces autophagy, revealing an inverse relationship between autophagy and apoptosis (Ferrara et al., 2023). Similarly, Caprine herpesvirus type 1 (CpHV-1) induces apoptosis and autophagy in various cell lines, primarily through the activation of caspase-3 and caspase-9, implicating the intrinsic pathway (Montagnaro et al., 2019). CpHV-1 infection also promotes autophagy, as evidenced by the enhanced conversion of LC3-I to LC3-II and the reduction of p62 protein levels in U2OS and A549 cells. This dual induction of apoptosis and autophagy suggests a complex interaction whereby CpHV-1 modulates cell death pathways to optimize its replication and dissemination. Equid Alphaherpesvirus 1 (EHV-1) similarly induces apoptosis and autophagy in infected neuronal cells. Our data further demonstrate an increase in reactive oxygen species (ROS) production, particularly at early stages of infection, correlating with mitochondrial damage and apoptotic signaling (Mesquita et al., 2021a). EHV-1 infection also triggers autophagy, as indicated by the elevated levels of LC3-II protein and the colocalization of viral antigens with LC3B, suggesting the formation of autophagosomes (Mesquita et al., 2021b). The induction of autophagy likely serves as a cellular response to mitigate damage caused by viral infection and maintain cellular homeostasis.

The intriguing capacity of PRV to regulate pathways such as ferroptosis and cuproptosis is noteworthy, especially considering the neurotropic properties of the virus. Ferroptosis, characterized by iron-dependent lipid peroxidation, and cuproptosis, associated with copper-mediated protein aggregation, are both implicated in neuronal cell death (Li et al., 2020; Fan et al., 2024; Fang et al., 2024). The inhibition of these pathways may serve as an adaptive strategy employed by PRV to maintain the structural and functional integrity of neuronal cells, thus facilitating a prolonged environment conducive to latent infection. This hypothesis is bolstered by the increased expression of genes such as Alox12 and Tfrc, potentially indicative of a compensatory mechanism aimed at regulating cellular iron levels and mitigating oxidative stress within the neuronal milieu (Pomeranz et al., 2005; Wu et al., 2020).

Our research investigates the notable metabolic reprogramming observed in cells infected with PRV. This shift in metabolism is believed to be a result of direct viral manipulation, with the goal of enhancing the supply of biosynthetic building blocks and energy necessary for viral replication. These changes not only support viral proliferation but may also play a role in the inhibition of cell death pathways, as alterations in metabolism can have a significant impact on cellular survival and programmed cell death (Laval et al., 2018). Despite the significant findings, this study has several limitations. The use of the Neuro-2a cell line, while valuable, may not fully capture the complexity of PRV interactions *in vivo*. Future studies should aim to validate these findings in primary neuronal cells and animal models to better understand the *in vivo* relevance of the identified pathways. Additionally, while our study highlights several key pathways manipulated by PRV, the precise molecular mechanisms remain to be fully elucidated. Further research should focus on dissecting these mechanisms and exploring the potential for targeted therapeutic interventions.

In conclusion, our transcriptomic analysis has elucidated the strategies employed by PRV to modulate host cell pathways, notably by suppressing various forms of programmed cell death to enhance its replication and dissemination within Neuro-2a cells. This study specifically contributes to the field by identifying key differentially expressed genes associated with necroptosis, pyroptosis, autophagy, ferroptosis, cuproptosis, and disulfidptosis pathways, thus providing a comprehensive overview of the viral manipulation of host cell death mechanisms. These insights not only deepen our understanding of PRV pathogenesis but also highlight potential molecular targets for therapeutic interventions aimed at disrupting PRV’s ability to evade host immune responses and sustain infection. The results offer a valuable framework for future studies on herpesvirus biology and the development of targeted antiviral therapies.

## Data Availability

All data generated and analyzed from this study are included in this published article. The raw RNA-Seq data has been submitted to NCBI Short Read Archive (SRA) under Bioproject PRJNA1137426 (https://www.ncbi.nlm.nih.gov/bioproject/PRJNA1137426/).

## Footnotes

^1^ http://www.r-project.org/

^2^ http://www.geneontology.org/

^3^ http://www.gsea-msigdb.org/gsea/msigdb/index.jsp

^4^ http://www.tanpaku.org/autophagy/list/GeneList.html#

^5^ http://www.zhounan.org/ferrdb
